# Implant Stability in the Posterior Maxilla: A Controlled Clinical Trial

**DOI:** 10.1155/2017/6825213

**Published:** 2017-05-25

**Authors:** Raquel Zita Gomes, Mario Ramalho de Vasconcelos, Isabel Maria Lopes Guerra, Rute Alexandra Borges de Almeida, Antonio Cabral de Campos Felino

**Affiliations:** ^1^Department of Oral Surgery, Faculty of Dental Medicine, University of Porto, Rua Manuel Pereira da Silva, 4200-393 Porto, Portugal; ^2^Department of Biomaterials, Faculty of Dental Medicine, University of Porto, Rua Manuel Pereira da Silva, 4200-393 Porto, Portugal; ^3^Mathematics Research Center, Statistics, Modulation and Computer Applications Office, Faculty of Sciences and Mathematics, University of Porto, Rua do Campo Alegre 687, 4169-007 Porto, Portugal

## Abstract

**Aim:**

To evaluate the primary and secondary stability of implants in the posterior maxilla.

**Methods:**

Patients were allocated into three groups: (A) native bone, (B) partially regenerated bone, and (C) nearly totally regenerated bone. Insertion torque (IT) and implant stability quotient (ISQ) were measured at placement, to evaluate whether satisfactory high primary stability (IT ≥ 45 N/cm; ISQ ≥ 60) was achieved; ISQ was measured 15, 30, 45, and 60 days after placement, to investigate the evolution to secondary stability.

**Results:**

133 implants (Anyridge®, Megagen) were installed in 59 patients: 55 fixtures were placed in Group A, 57 in Group B, and 21 in Group C. Fifty-two implants had satisfactory high primary stability (IT ≥ 45 N/cm; ISQ ≥ 60). A positive correlation was found between all variables (IT, ISQ at* t* = 0,* t* = 60), and statistically higher IT and ISQ values were found for implants with satisfactory high primary stability. Significant differences were found for IT and ISQ between the groups (A, B, and C); however, no drops were reported in the median ISQ values during the healing period.

**Conclusions:**

The evaluation of the primary and secondary implant stability may contribute to higher implant survival/success rates in critical areas, such as the regenerated posterior maxilla. The present study is registered in the ISRCTN registry with ID ISRCTN33469250.

## 1. Introduction

Dental implants are considered the best treatment option to replace nonrestorable or lost teeth: both professionals and patients are now convinced of the validity of this treatment procedure that shows reliable long-term results [1, 2].

In the posterior maxilla, the rehabilitation of patients with implant supported fixed partial prostheses or single crowns is now a safe and effective procedure, as demonstrated by several clinical studies [3, 4].

In cases of advanced or severe vertical and/or horizontal bone defects, regenerative surgical techniques are essential to correct the initial anatomical situation, to allow the proper placement of dental implants in the posterior maxilla [5].

Among these regenerative techniques, maxillary sinus augmentation [3–7], guided bone regeneration (GBR) [8], and split-crest techniques [9] are the most commonly used procedures to restore the ideal anatomical bone conditions and to allow simultaneous and/or subsequent placement of dental implants.

However, in recent years, it has been shown that the success of fixed rehabilitation with dental implants depends not only on the quantity (volume) of bone available for implant placement, but also on the quality of this bone [10, 11].

The assessment of the quality of the bone structure should be considered essential, prior to implant placement [11, 12]. In fact, the achievement and maintenance of an adequate implant stability are fundamental prerequisites for the long-term positive outcomes of osseointegrated implants [13, 14]. Implant stability is key for clinical success [13, 14]. The concept of primary stability is related to the lack of mobility of the implant after its placement in the implant bed [13–18] and to the measurement of the consistency of bone/implant complex [15–20]. There are two types of implant stability: primary and secondary stability [18–23].

Primary stability comes from the mechanical engagement of the fixture with cortical bone [13–18]. It is determined by the quantity and quality of the available bone at implant placement, but also from the surgical procedure [10, 15, 16] and from the dimensions (length, diameter) and design (macrotopographical features) of the fixture [3, 4, 8, 10, 11].

Secondary stability comes from regeneration and remodeling of the bone and tissue around the implant after insertion, and it mainly depends on the micro/nanotopographical features of the implant [13–18, 22–24]; however, it seems to be highly dependent on the primary stability [10, 22, 23].

In modern implantology, with the introduction of surgical and prosthetic protocols such as the immediate placement of implants in fresh postextraction sockets [19, 24–26] and immediate functional loading [19, 20, 27], it is very important to quantify implant stability at various timeframes, in order to have a long-term prognosis for the implants placed [10, 14–18]. Therefore, the employment of simple, clinically applicable noninvasive tests to assess implant stability and osseointegration can be considered valuable [12–18, 23]. So far, several nondisruptive methods have been suggested to evaluate the implant stability, such as percussion tests, radiography, insertion torque (IT) measurement [12–18], and resonance frequency analysis (RFA) with the implant stability quotient (ISQ) scale [12–18, 23]. Nowadays, the most objective and commonly used tests to measure primary stability are IT and RFA by means of ISQ measurements: the IT measures the rotational stability while the ISQ measures the axial stability of the implant [12, 18–23]. However, only the ISQ allows for the monitoring of the evolution of the fixture stability during healing time, from primary to secondary implant stability [13–18]. The ISQ, ranging from 1 to 100 and measured with RFA, is a scale indicating the stability of dental implants [4, 12–19, 23]. At placement, the stability is considered acceptable if comprised between 55 and 85 ISQ [4, 12–19, 23], and higher values are generally found in the mandible rather than the maxilla [4, 12–19, 23]. In all cases, a massive decrease in ISQ values indicates a potential problem at the bone-implant interface, and it should be considered as an early warning because it can be indicative of clinical problems that can lead to implant failure [12, 18–23]. In the first healing period, the implant stability usually slightly decreases, because remodeling processes affect the preexisting bone, responsible for the initial mechanical stabilization. Later, however, the stability of the implant tends to increase with time, enforced by the new bone apposition onto the implant surface and the establishment of a secondary, biological stability [13, 16–23]; after the bone healing is completed, the average ISQ value of all implants is approximately 70 [12–18]. Consequently, if the ISQ at placement is sufficiently high, a small drop in stability during the initial healing phase has no clinical consequences; however, in the event of rather low ISQ values at placement, a decrease of stability may represent a problem, posing a risk for implant survival [4, 12–18, 23].

In the posterior maxilla, the bone quality is lower than in the mandible [3–7, 9–11, 24–27], particularly in the case of partially or nearly totally regenerated bone [3–7, 9, 11, 24].

Therefore, the aim of the present controlled clinical trial is to evaluate the stability of dental implants at placement, in the human posterior maxilla, and to investigate the evolution from primary to secondary stability, in three different groups: patients with native bone, patients with partially regenerated bone, and patients with nearly totally regenerated bone.

## 2. Materials and Methods

### 2.1. Patient Selection

The sample for the present study came from subjects referred for dental implant treatment to a single private clinical centre (Clinica Médico-Dentária RZG), over a two-year period (2012–2014). The study was performed in collaboration with the Department of Oral Surgery, Faculty of Dental Medicine, University of Porto. The inclusion criteria were all adult patients (age > 18 years) in good medical and oral conditions, who needed one or more dental implants in the posterior maxilla, for supporting fixed rehabilitations (single crowns or fixed partial prostheses). The willingness to fully participate in the study, attending all the requested follow-up sessions, was also an inclusion criterion. Exclusion criteria included patients with uncontrolled systemic diseases (uncompensated oral diabetes), patients with a history of head/neck irradiation, patients with hemophilia, patients with immune system severe deficiencies, and patients under pharmacological therapies that could alter bone metabolism (patients treated with oral/intravenous amino bisphosphonates). Pregnancy and lactation were also exclusion criteria. Smoking habit was not an exclusion criterion per se, as only patients smoking >20 cigarettes/day (heavy smokers) were excluded from the present investigation. Conversely, bruxism was not an exclusion criterion for this study. Finally, patients who did not attend the final required follow-up session (2 months after implant placement) had to be excluded from the statistical evaluation. All patients were fully informed of the nature of the present study and signed an informed consent form for the implant treatment. The study was approved by the Ethics Committee of the Faculty of Dental Medicine of the University of Porto (process number #890573) with the title “Controlled Clinical Trial about the Effects of Bone Regeneration in the Implant Stability during the Healing Phase.” All the procedures followed the standards of the Helsinki Declaration of 1975, as revised in 2000.

### 2.2. Preoperative Evaluation

Before implant placement, each patient was investigated clinically and radiographically. Panoramic and periapical radiographs were the primary radiographic investigations, followed by a cone beam computed tomography (CBCT) scan requested by the surgeon. CBCT was used to accurately assess, in three dimensions (3D), the bone volume (height/width) available for implant placement. CBCT data could be imported and loaded into specific navigation software (R2Gate®; MegaGen Implant, Gyeongbuk, South Korea), with the aim of performing a 3D reconstruction of the edentulous areas; it was therefore possible to correctly assess the height/width of each implant site and the thickness/density of the cortical plates and cancellous bone, as well as the ridge angulations. The preoperative evaluation included stone casts and diagnostic wax-up.

### 2.3. Study Design

In order to ensure that the comparisons were performed between implants in the same location, the implant locations were restricted to the posterior maxilla (premolar and molar areas). The eligible patients were allocated (divided) into three different groups, corresponding to three different clinical situations.

*(i) Group A: Nonregenerated (NR), Native Maxillary Bone*. This group consisted of patients who had not received any regenerative procedure in the posterior maxilla. In these patients, therefore, the implants were placed in entirely native, nonregenerated bone.

*(ii) Group B: Partially Regenerated (PR) Maxillary Bone*. This group consisted of patients with 3 to 6 mm of available bone in the posterior maxilla, who had been treated with GBR, with the aim of vertically/horizontally augmenting the bone volume available for implant placement. In these patients, therefore, the implants were mainly placed in native bone, but a certain amount of regenerated bone was also present.

*(iii) Group C: Nearly Totally Regenerated (TR) Maxillary Bone*. This group consisted of patients with less than 3 mm of remaining native bone in height, who had been subjected to maxillary sinus augmentation with at least 8 months of healing time prior to implant placement. In these patients, therefore, the implants were mainly placed in regenerated bone.

The allocation of the patients in the three groups was based on the patients' anamnesis and clinical and radiographic evaluation; the size and distribution of the sample were established as a minimum of 15 to 20 implants per group. Ideally, the distribution between groups should be balanced. Implants in the same patient could belong to different groups and were always considered as independent. The clinical study was performed over a two-year (2012–2014) period. The follow-up after implant placement was after 2 months.

### 2.4. Dental Implants

The tapered fixtures used in the present trial (Anyridge, Megagen, Gyeongsang, South Korea) are characterized by a peculiar macrotopography, with a knife-edge (KnifeThread®, Megagen, Gyeongsang, South Korea) thread design. This design can guarantee high primary stabilization, even in difficult clinical contexts, such as in the case of low bone quality/density [4, 8], in postextraction sockets [19], or under immediate loading protocol [19, 20]. The surface of these implants was the result of a sandblasting treatment (resorbable blast media) and the subsequent incorporation of calcium ions by means of a hydrothermal treatment [28]. This nanotopographical surface (Xpeed®, Megagen, Gyeongsang, South Korea) is characterized by increased surface area/energy, for a better interaction with biological fluids, and therefore has the potential to stimulate and accelerate osseointegration [28, 29]. Finally, from the prosthetic point of view, such fixtures possess a 5 mm deep conical connection (10°) combined with an internal hexagon, capable of ensuring high mechanical stability and a suitable biological seal; an integrated switching platform is present, to maintain peri-implant tissue volume over time [19, 20]. The implants were available in various lengths (7.0, 8.5, 10.0, 11.5, 13.0, and 15.0 mm) and diameters (3.5, 4.0, 4.5, 5.0, 5.5, and 6.0 mm) depending on the surgical requirements.

### 2.5. Implant Placement

**Figure 1:**
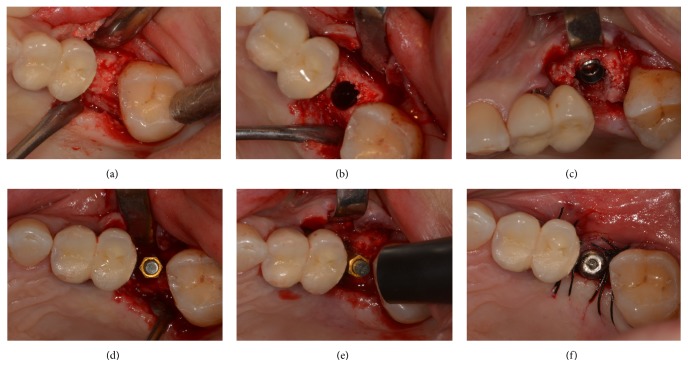
Implant placement and ISQ measurement in a patient with partially regenerated bone (Group B): (a) a mucoperiosteal flap is raised and the partially regenerated bone is exposed; (b) the implant site is prepared in the partially regenerated bone; (c) the implant is placed in the partially regenerated bone; (d) the Smartpeg is connected for ISQ measure; (e) ISQ measure at placement; (f) since a satisfactory high primary implant stability is achieved (IT ≥ 45 N/cm and ISQ ≥ 60), the implant is not submerged and the transmucosal healing abutment is placed.

The study was conducted by a single implantologist (RZG) with extensive experience, who performed all the surgeries. Implant placement was performed using a conventional surgical protocol, with the elevation of a mucoperiosteal flap in all treatment groups. The surgery involved infiltrating local anesthesia with articaine 2% plus epinephrine 1 : 100,000, linear incision in the bone crest, elevation of the mucoperiosteal flap, and bone drilling according to the manufacturer's recommended protocols. Finally, implant placement with geared motor through direct mechanical implantation or calibrated torque wrench carrier was carried out. The implants were positioned slightly subcrestally (0.5 to 1 mm), according to the manufacturer's recommendation (Figures 1(a), 1(b), and 1(c)). Lastly, cover screws or healing abutments were placed. The conditions for placement of a healing abutment (transmucosal healing of the implant) were IT ≥ 45 N/cm and ISQ ≥ 60 at placement (i.e., a satisfactory high implant stability at placement). In the event that this circumstance was not achieved, the implants were submerged; these fixtures were left submerged for a period of healing of 60 days; following this, second-stage surgery was performed to uncover them and obtain ISQ measure at 60 days. In all procedures, the grafting heterologous materials used were particulate prehydrated bone (Osteobiol mp3®, Tecnoss, Turin, Italy) and collagen membranes (Osteobiol Evolution®, Tecnoss). Flaps were sutured with coated multifilament polyamide 4(0) simple stitches.

### 2.6. Outcome Variables

#### 2.6.1. Insertion Torque (IT)

The insertion torque (IT) of each implant was assessed at the time of implant placement (insertion) with a surgical motor (Bien-Air®, Bien-Air MT, Bienne, Switzerland)  with 20 : 1 reduction and/or with a calibrated manual torque wrench. The unit of measure for IT was newtons per centimeter (N/cm).

#### 2.6.2. Implant Stability Quotient (ISQ)

Resonance frequency analysis (RFA) was employed to measure implant stability with a dedicated instrument (Osstell Mentor®; Osstell, Integration Diagnostics, Sweden). This portable device emits magnetic pulses to a small magnet (Smartpeg®) screwed directly onto the implant with 5 Ncm; the magnet starts to vibrate, and the probe listens to the tone and translates it to an implant stability quotient (ISQ) value [12, 13, 17, 19, 23]. For each implant, ISQ values (scaled 1–100) were measured from the four sites (mesial, distal, buccal, and palatal sites). The mean of all measurements was rounded to a whole number and regarded as the final ISQ of the implant [19, 23]. RFA measurements were performed immediately after implant placement (Figures 1(d), 1(e), and 1(f)) and then after 15, 30, and 45 days in cases in which satisfactory primary stability was successfully achieved and after 60 days in all cases. These intervals were chosen in order to investigate the progression of ISQ during healing time, until complete bone healing. The first (ISQ at time 0) and the last (ISQ at day 60) ISQ measures were mandatory for the inclusion of the patient in the statistical analysis, while the intermediate measures of ISQ (at 15, 30, and 45 days) were not considered mandatory for the inclusion in the statistical evaluation (due to the possible lack of compliance of some patients, who could miss one or more intermediate controls, or due to the fact that implants were left submerged for two months, because of a lack of satisfactory primary stability at placement).

### 2.7. Statistical Evaluation

The responsible units for the statistical analysis were the Centro de Matemática da Universidade do Porto (CMUP) (Mathematics Centre of Porto University) and the GEMAC (Gabinete de Estatistica, Modelação e Aplicações Computacionais) (Statistics, Modulation and Computer Applications Office). Statistical analysis was performed using R Project for Statistical Computing software® (GNUS). The Shapiro-Wilk test was considered for Gaussianity testing and particular nonparametric tests were preferred to avoid significance loss related with no Gaussianity of some variables. Descriptive statistics were used to evaluate the study sample (the distribution of patients and implants). According to the preestablished protocol, the implants were considered satisfactorily stable only with IT ≥ 45 N/cm and ISQ ≥ 60 at placement; conversely, with IT < 45 N/cm and/or ISQ < 60 at placement, the implants were considered not satisfactorily stable at placement.

After that, the following statistical evaluations were performed:

- Minimum, 1st quartile, median, mean, 3rd quartile, and maximum values were calculated for IT and ISQ at placement and 2 months later.
- The linear dependence between IT and initial and final ISQ was evaluated using an association rank *t*-test for paired samples over Spearman's correlation coefficient, over all implants and considering only implants that were highly stable at placement.
- The implants that were highly stable at placement were also compared with the other implants (implants with IT < 45 N/cm and/or ISQ < 60 at placement, i.e., no satisfactory high stability at placement) using the Mann-Whitney-Wilcoxon test.
- Multiple comparisons between the measures in the three different levels of osseous regeneration (A, B, and C) were compared by Kruskal-Wallis rank sum *t*-testing, separately for implants with and without satisfactory stability at placement (IT and initial and final ISQ).
- For the implants with stability at placement, the Friedman test was considered to evaluate the differences in the median ISQ measures with healing time across heterologous osseous regeneration and measures with osseous regeneration across healing time.

A pairwise Wilcoxon test with Bonferroni correction was performed for post hoc testing. In all hypothesis testing, statistical significance was assumed for *p* value less than 0.05 (proof test value < 5%).

## 3. Results

The study sample included 137 implants placed in 60 patients (23 males, 37 females). The percentage of female patients (61.7%) was higher than that of male patients (38.3%). The mean age of patients was 56.18 ± 11.76 years and the median was 56 years. Ninety-one implants were placed in females whereas 46 implants were placed in males. However, the eligible data for statistical analysis was of 59 patients with 133 implants, as cases for which final ISQ at 60 days was missing were not considered in this analysis (one patient, four implants). Of these four missing values, two were failed implants, one was an implant placed in native bone (Group A), and the other was a fixture inserted in partially regenerated bone (Group B). At the end, the survival rate of the implants placed in this study was 98.52%, with 133 surviving implants over 135. With regard to the different groups, 55 implants were placed in Group A (native bone), 57 implants were inserted in Group B (partially regenerated bone), and 21 implants were placed in Group C (almost totally regenerated bone): the number of observations per variable, per group (level of osseous regeneration), is summarized in Table 1. Satisfactory high initial stability conditions were defined as an IT ≥ 45 N/cm and ISQ ≥ 60 at placement. Of the 133 implants inserted here, 52 (52/133: 39.1%) had satisfactory high primary stability according to the established protocol, and 81 (81/133: 60.9%) did not. From the 52 implants with satisfactory high primary stability, 25 were from Group A (25/55: 45.5%), 20 from Group B (20/57: 35.1%), and 7 from Group C (7/21: 3%). With regard to implants with satisfactory high primary stability, the number of observations per variable, per group (level of osseous regeneration), is summarized in Table 2. Conversely, 81 implants had no satisfactory high stability at placement (they had IT < 45 N/cm and/or ISQ < 60 at placement): according to the established treatment plan, these implants were left submerged for a period of healing of 60 days, so they did not have ISQ measurements at 15, 30, and 45 days. From the 81 implants without satisfactory high primary stability, 30 were from Group A (30/81: 37.0%), 37 from Group B (37/81: 45.7%), and 14 from Group C (14/81: 17.3%).

**Figure 2:**
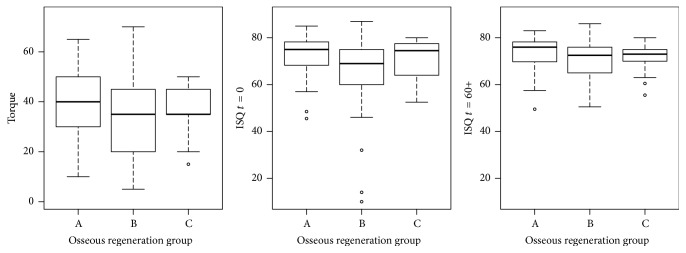
IT and ISQ at *t* = 0 and *t* = 60 values distributions per group (levels of osseous regeneration) for all the 133 implants in the study. Group A: nonregenerated (native) bone; Group B: partially regenerated bone; Group C: nearly totally regenerated bone.

**Figure 3:**
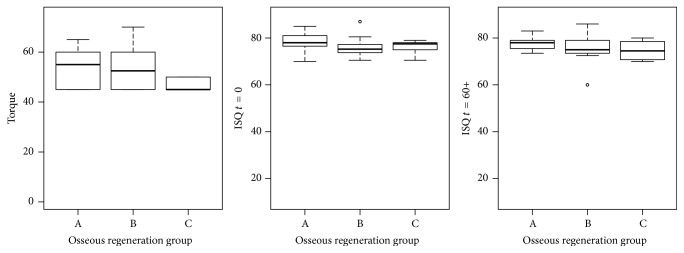
IT and ISQ at *t* = 0 and *t* = 60 values distributions per group (levels of osseous regeneration) for implants with satisfactory high initial stability (IS) according to the protocol. Group A: nonregenerated (native) bone; Group B: partially regenerated bone; Group C: nearly totally regenerated bone.

**Figure 4:**
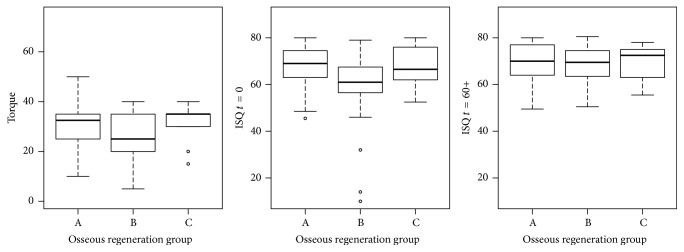
Torque and ISQ at *t* = 0 and *t* = 60 values distributions per group (levels of osseous regeneration) for implants without satisfactory high initial stability (WIS) according to the protocol. Group A: nonregenerated (native) bone; Group B: partially regenerated bone; Group C: nearly totally regenerated bone.

The minimum, 1st quartile, median, mean, 3rd quartile, and maximum values of each variable (IT and ISQ at placement, ISQ at 60 days) for all implants, for implants with satisfactory high primary implant stability, and for implants without primary stability according to the protocol are presented in Table 3. The IT and ISQ (*t* = 0 and *t* = 60) values per group are presented in Figure 2 (all implants), Figure 3 (implants with satisfactory high primary implant stability (IS)), and Figure 4 (implants without satisfactory high primary stability (WIS)). In those plots, the central box goes from the 1st to the 3rd quartile, the median is marked as a horizontal line, whiskers connect to the maximum and minimum values, and outliers are excluded, marked as circles and defined as values out of the 1.5 of the interquartile range. Both IT and ISQ measures achieved statistically higher values for cases with satisfactory high primary stability, as those measures are the base of such aggrupation. Group C in cases of initial stability presented lower IT values.

A linear dependence between variables (evaluated using an association rank for paired samples, over Spearman's correlation coefficient) was present, considering all implants (Table 4) and implants with satisfactory high stability at placement (Table 5). A positive correlation was observed between all variables, and all values were highly significant, for all implants and for the group of implants with satisfactory high stability at placement.

The Mann–Whitney test suggested significant differences (*p* < 0.005, *p* < 10^−7^) between the relative locations for cases with and without satisfactory high initial stability, for all measures available (IT and ISQ, *t* = 0 and *t* = 60). Considering all data, the Shapiro-Wilk test allowed the rejection (*p* < 0.05) of the hypothesis of Gaussian distribution for IT measures and both initial and final ISQ values. Considering separately the initial stability level (with or without), the same conclusion was obtained for all, except IT, in cases with initial stability (*p* value ≥ 0.05408) and ISQ at 60 days (*p* value ≥ 0.176). Thus, for the remaining of the analysis, the normality of the variables was not assumed and nonparametric tests were preferred.

IT and ISQ measures were compared for Groups A, B, and C using the Kruskal-Wallis rank sum* t*-test using two separate groups, for implants with and without satisfactory high primary stability, respectively. Significant differences between the distributions of the levels of osseous regeneration were found for IT in the case of initial stability between Groups A and C and B and C, but not between A and B. For implants without initial stability, statistically significant differences were found between Groups B and C. No significant differences were found between the distributions of the levels of osseous regeneration for ISQ at *t* = 0 in cases with satisfactory primary stability, while in the cases without satisfactory initial stability, differences between Groups A and B were found (post hoc tests). Also, no significant differences were found at *t* = 15, *t* = 30, *t* = 45, and *t* = 60, in the case of initial stability. Only in the cases without satisfactory high primary stability were statistically significant differences found in the ISQ at *t* = 0 between Group A and Groups B and C (not between Groups B and C), but not for ISQ at *t* = 60. The median values of ISQ measures at each time per Group A, B, or/and C were calculated for cases with initial stability, ignoring missing values, obtaining a completely balanced design, the results of which are presented in Table 6.

**Figure 5:**
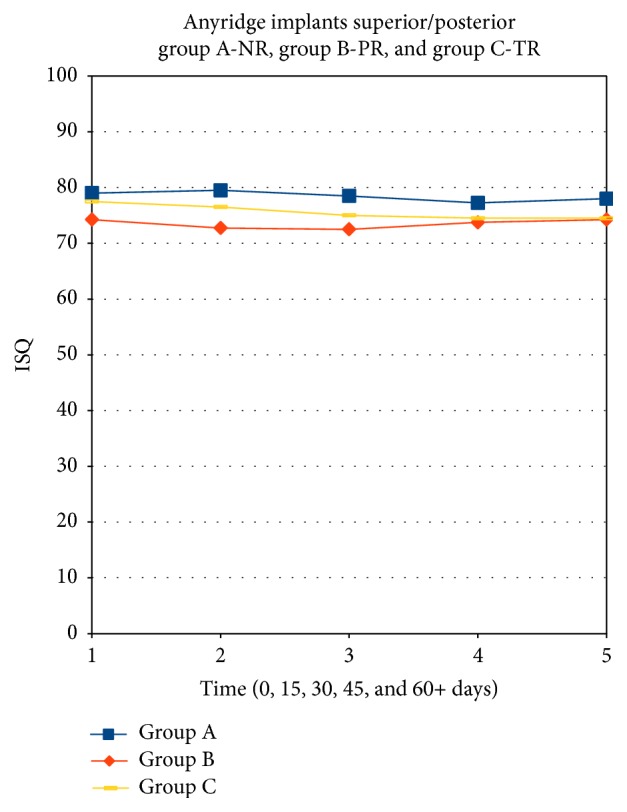
Progression of ISQ values during the healing phase in the 3 groups of patients (Group A: nonregenerated bone; Group B: partially regenerated bone; Group C: nearly totally regenerated bone).

No significant differences were found between median ISQ at different times, considering the level of osseous regeneration as a grouping factor using the Friedman test (*p* ≥ 0.40). Significant differences were found between the median ISQ for the 3 levels of osseous regeneration, considering time as a grouping factor using the Friedman test (*p* < 0.05). A post hoc test with Bonferroni correction found statistically significant differences (*p* < 0.05) between Group A and Groups B and C. On the other hand, no differences between Groups B and C were found. No drops were found in ISQ values across time (Figure 5), in the different groups.

## 4. Discussion

When an implant is placed in the posterior maxilla, it can be difficult to obtain a high and satisfactory primary stability, compatible with an immediate functional loading protocol: in fact, the bone quality (density) is rather poor in this region, as well as in the case of native bone [3–7, 9, 11, 24–27]. When the bone has been partially or fully regenerated, the bone quality tends to further decrease [3–7, 9–11, 24], and the immediate loading can become even more dangerous.

Since the primary stability is such an important element and can determine the success or failure of implant therapy, many authors have studied it [12–19, 30–35]. Primary implant stability can be evaluated at baseline either through IT registration or through ISQ assessment [12–19, 30–35]. Maximum torque of the implant placement provides the rotational stability of the implant. Conversely, the ISQ assesses the axial stability of the implant in different directions [12–19, 30–35]. This postsurgical information is both objective and complementary, allowing for the best clinical decision to be made regarding the several possibilities of surgical and prosthetic protocols. Furthermore, the ISQ allows noninvasive monitoring over time while IT can be measured only once [12–19, 32, 34].

Several authors have suggested that primary stability may be a useful predictor for osseointegration [29–35] and that a high primary stability makes immediate loading more predictable [19, 20, 26, 27]. Park et al. showed with an animal experimental model that ISQ values have a significant correlation with Bone-Implant Contact (BIC) percentage [30]. Meanwhile, Rodrigo et al. demonstrated that the evaluation of RFA values (ISQ) had a statistically significant correlation with implant outcome [32]. In fact, in that study, no implant with ISQ > 60 failed, while 19% of implants with ISQ < 60 failed [32]. Pagliani and collaborators further proved that the correlation between micromobility of implants and ISQ is nonlinear and micromotion is reduced by approximately 50% from 60 to 70 ISQ [33], while Turkyilmaz et al. found a positive strong correlation between bone density (calculated with computed tomography) and IT/ISQ insertion torque, as well as a positive correlation between IT and ISQ [34]. Degidi et al. found statistically superior ISQ values for implants inserted in grated sinus compared with postextraction implants, at placement [35]. Degidi et al. also compared healed sites with augmented sinus and found statistically significant differences between these two groups, with higher ISQ values in augmented sinus at implant placement [35]. However, in that study, the authors did not provide any information about the ISQ values during or after the healing period [35].

In the present clinical study, the primary stability was evaluated as well as its evolution to secondary stability in implants placed in the posterior maxilla, in native, partially regenerated, and (almost) totally regenerated bone, respectively. Overall, 133 fixtures have been included, placed in 59 patients: the primary stability of these fixtures was recorded, as well as their evolution into secondary stabilization, in the first period of healing, using two separate indices: the insertion torque (IT) and the implant stability quotient (ISQ), with measurements up to 60 days. With regard to primary stability and in reference to the criteria adopted in the present work, 52 implants (39.1%) were placed with a high primary stability (IT ≥ 45 N/cm and ISQ ≥ 60) and 81 (60.9%) were placed without a high primary stability. In particular and in reference to the aforementioned three groups of patients, 55 implants were placed in native bone: of these, 25 (45.5%) had a high primary stability whereas 30 (54.5%) did not. Fifty-seven implants were placed in partially regenerated bone through guided bone regeneration (GBR) techniques: of these, 20 showed high primary stability at insertion (35.1%) and 37 (64.9%) did not. Finally, only 7 (33.3%) of the implants inserted into the almost completely regenerated bone (via sinus lift) showed high stability at placement; in contrast, 14 implants (66.7%) did not have high stability at insertion.

These data are important because they substantially confirm what was reported in the current literature [4, 10, 11, 13–16, 20]. In fact, in the present study, higher IT/ISQ values in implants with satisfactory high primary stability were found, as well as a positive linear correlation between the selected variables (IT and ISQ) for all implants and for implants with satisfactory high primary stability.

Obviously, significant differences were also found in the level of implant stability between the three groups of patients (native, partially regenerated, and almost totally regenerated bone): this confirms that the presence of regenerated bone can negatively affect the primary stabilization of the fixture (the percentage of the fixtures with satisfactory high primary stability in this study tended to decrease, from the group of patients with native bone towards the group of patients with totally and partially regenerated bone), as reported in the literature [4, 10, 11, 13–16, 20].

However, it must be pointed out that the mean values of primary stability obtained in the difficult clinical context of our present study were quite high. This result was certainly determined by the careful insertion protocol followed, with underpreparation of surgical sites [15, 16, 19, 20]; but it was also determined by the macrotopographical characteristics of the implants used [19, 21]. In fact, in the present work, tapered implants have been used with knife-edge threads [4, 19, 20]. The body of this fixture is narrower than the threads, and the threads are extremely aggressive; this shape may provide a better anchorage to bone, thus better primary stabilization [4, 19, 20], even in difficult cases such as partially [8] or nearly totally [4] regenerated bone, or in postextraction sockets [19, 20]. Previous studies have already demonstrated that tapered implants tend to have higher ISQ values than cylindrical ones [18, 21]; the presence of a thread with this accentuated design could be a further positive element, to facilitate the stabilization of fixture in difficult situations [4, 8, 19, 20].

Moreover, another very important element emerging from this study is that, in the three different groups of patients, no decrease/drop in the stability values (ISQ) was found over time, in the first healing period. This finding is in contrast with what is reported in the literature [12–18, 31–34]. Other studies, in fact, reported a drop in the ISQ values in the healing phase (after 2 to 6 weeks of the implantation) [12–18, 31–34]. In our present study, the aforementioned drop was not observed and the ISQ values stayed very stable in the first two months of the healing time (the most critical phase for implant stabilization). This result is the most important element emerging from the study, since the absence of drop in the implant stability during the first healing period may potentially contribute to higher implant survival/success rates in critical areas, such as the regenerated posterior maxilla. This excellent result certainly depends in the first place on the high primary stability obtained at implant placement: in fact, this study has shown that there is a linear correlation between the values of IT and ISQ at insertion and ISQ during the healing period, for all the fixtures and for the fixtures with satisfactory high primary stability. However, this evidence could also be related to the surface of the implants used in the present study [4, 20]. In fact, the implants used had a novel nanostructured calcium-incorporated surface, which could have the ability to accelerate bone healing, as demonstrated in recently published human histologic studies [28, 29].

Obviously, the present study has limitations, such as the limited number of patients treated and fixtures inserted; in particular, only a few implants were inserted in Group C (nearly totally regenerated bone), and this is a major limitation of the present work, since Group C was probably the most interesting to investigate, and it would have been appropriate to have inside it a higher number of fixtures. Another limitation of the present study is the short follow-up: the implants were controlled 2 months after placement and a part of them (those that did not have satisfactory high primary stability at placement) were submerged, so they did not have ISQ measurements at 15, 30, and 45 days because it was impossible to connect the Smartpeg to measure the ISQ. Further, long-term controlled studies are therefore needed to confirm the outcomes emerging from the present work.

## 5. Conclusions

In the present study, 133 implants were installed in 59 patients in the posterior areas of the maxilla of three different types of patients: patients with native bone (Group A, 55 implants), patients with partially regenerated bone (Group B, 57 implants), and patients with nearly totally regenerated bone (Group C, 21 implants). The primary implant stability was measured at placement, by means of insertion torque (IT) and implant stability quotient (ISQ). According to a preestablished protocol, an implant was considered satisfactorily stable with IT ≥ 45 N/cm and ISQ ≥ 60; conversely, in case of IT < 45 N/cm and/or ISQ < 60, the implant was not considered satisfactorily stable. After that, the evolution from primary to secondary implant stability was studied, by means of ISQ, at different times (15, 30, 45, and 60 days after placement for implants with satisfactory high primary stability and 60 days for implants without it). Fifty-two implants had satisfactory high primary stability, according to the established protocol (IT ≥ 45 N/cm, ISQ ≥ 60), and 81 did not. A positive correlation was observed between all variables (IT, ISQ at *t* = 0, *t* = 60), indicating linear relation, and statistically higher IT and ISQ values were found for implants with satisfactory high primary stability, when compared with implants without satisfactory high primary stability. Statistically significant differences were found for IT and ISQ value between the different groups: A, B, and C; however, no drops were reported in the median ISQ values during the healing period. With regard to this last finding, the implant system used in this study showed linear ISQ progression, without a significant drop of stability values within the first two months of healing. This last finding may be related to the surgical protocol adopted, but also to the macrotopographical features (threads design) and to the surface of the implant used in this study, characterized by a peculiar nanotopographic design.

## Figures and Tables

**Table 1:** Number of observations (per variable and per group) in all 133 considered implants.

Group	All implants (IT, initial and final ISQ measures)	Implants with ISQ measure at 15 days	Implants with ISQ measure at 30 days	Implants with ISQ measure at 45 days
A	55	14	14	10
B	57	14	13	11
C	21	12	12	12

Total	133	40	39	33

**Table 2:** Number of observations (per variable and per group) in the 52 implants with satisfactory primary implant stability (IT ≥ 45 N/cm and ISQ ≥ 60).

Group	Implants with satisfactory primary stability (IT, initial and final ISQ measures)	Implants with satisfactory primary stability with ISQ measure at 15 days	Implants with satisfactory primary stability with ISQ measure at 30 days	Implants with satisfactory primary stability with ISQ measure at 45 days
A	25	12	12	8
B	20	13	12	10
C	7	7	7	7

Total	52	32	31	25

**Table 3:** Statistical measures for all the implants and for implants with satisfactory primary implant stability (IS) (IT ≥ 45 N/cm and ISQ ≥ 60), in which ISQ measures were taken 15, 30, and 45 days after placement too.

Variable	*N *	Min.	1st quar.	Median	Mean	3rd quar.	Max.
IT	133	5.00	25.00	35.00	37.62	50.00	70.00
ISQ *t* = 0	133	10.00	63.00	72.50	69.06	77.00	87.00
ISQ *t* = 60	133	49.50	67.50	73.50	71.80	77.50	86.00
IT (IS)	52	45.00	45.00	50.00	52.50	60.00	70.00
ISQ *t* = 0 (IS)	52	70.00	75.00	77.25	77.03	79.00	87.00
ISQ *t* = 15 (IS)	32	59.50	72.88	76.25	75.58	79.12	85.50
ISQ *t* = 30 (IS)	31	59.50	72.50	76.00	75.35	79.25	87.00
ISQ *t* = 45 (IS)	25	60.00	72.50	75.50	75.44	75.44	86.00
ISQ *t* = 60 (IS)	52	60.00	74.50	77.50	76.57	79.00	86.00

**Table 4:** Correlation coefficients (Spearman), with *p* value between parenthesis, considering all implants.

All implants	IT	ISQ *t* = 0	ISQ *t* = 60
IT	1	—	—
ISQ *t* = 0	0.76 (2.2*∗*10^−16^)	1	—
ISQ *t* = 60	0.67 (2.2*∗*10^−16^)	0.70 (2.2*∗*10^−16^)	1

**Table 5:** Correlation coefficients (Spearman), with *p* value between parenthesis, considering implants that had satisfactory primary implant stability (IS).

Implants with satisfactory primary implant stability (IS)	IT	ISQ *t* = 0	ISQ *t* = 60
IT	1	—	—
ISQ *t* = 0	0.43 (0.001)	1	—
ISQ *t* = 60	0.46 (0.0005)	0.69 (1.5*∗*10^−8^)	1

**Table 6:** Median values of ISQ measures at each time, per group (Group A: nonregenerated bone; Group B: partially regenerated bone; Group C: nearly totally regenerated bone), in implants with satisfactory high primary implant stability.

	ISQ *t* = 0	ISQ *t* = 15	ISQ *t* = 30	ISQ *t* = 45	ISQ *t* = 60
A	78.00	79.25	77.75	78.50	78.00
B	75.25	73.50	74.00	73.75	75.00
C	77.50	76.50	73.50	74.50	74.50
